# Cardiac Toxicities in Oncology: Elucidating the Dark Box in the Era of Precision Medicine

**DOI:** 10.3390/cimb45100526

**Published:** 2023-10-15

**Authors:** Younan Samuel, Aswin Babu, Foteini Karagkouni, Ayden Ismail, Sunyoung Choi, Stergios Boussios

**Affiliations:** 1 Department of Cardiology, Medway NHS Foundation Trust, Windmill Road, Gillingham ME7 5NY, Kent, UK; younan.samuel@nhs.net (Y.S.); aswin.babu1@nhs.net (A.B.); foteini.karagkouni@nhs.net (F.K.); 2 GKT School of Medicine, King’s College London, London SE1 9RT, UK; ayden.ismail@kcl.ac.uk; 3 Department of Cardiology, Hampshire Hospitals NHS Foundation Trust, Aldermaston Road, Basingstoke RG24 9NA, Hampshire, UK; sunyoung.choi@kcl.ac.uk; 4 Department of Medical Oncology, Medway NHS Foundation Trust, Windmill Road, Gillingham ME7 5NY, Kent, UK; 5 Faculty of Life Sciences & Medicine, School of Cancer & Pharmaceutical Sciences, King’s College London, London SE1 9RT, UK; 6 Kent Medway Medical School, University of Kent, Canterbury CT2 7LX, Kent, UK; 7 AELIA Organization, 9th Km Thessaloniki—Thermi, 57001 Thessaloniki, Greece

**Keywords:** cardiotoxicity, chemotherapy, immunotherapy, targeted therapy, heart failure, cardiovascular disease

## Abstract

Despite current advancements in chemotherapy, immunotherapy and targeted treatments, the potential for major adverse cardiovascular events, regardless of previous cardiac history, persists. Scoring systems, such as the Heart Failure Association-International Cardio-Oncology Society (HFA-ICOS) risk assessment tool, can be utilized to evaluate several factors including prior cardiac history, risk factors and cardiac biomarkers to categorize patients into low, moderate, high, and very high-risk groups. Common cardiotoxicity complications include new or worsening left ventricular ejection fraction (LVEF), QT interval prolongation, myocardial ischaemia, hypertension, thromboembolic disease, cardiac device malfunction and valve disease. Baseline electrocardiogram (ECG) and transthoracic echocardiogram (TTE) are routinely performed for all patients commenced on cardiotoxic treatment, while other imaging modalities and biochemical markers have proven useful for monitoring. Management mainly includes early risk stratification and prompt identification of cardiovascular complications, with patient-specific surveillance throughout treatment. A multidisciplinary approach is crucial in determining the relationship between potential treatment benefits and cardiotoxicity, and whether the continuation of treatment is appropriate on a case-by-case basis. Early risk stratification, optimizing the patient’s cardiovascular status prior to treatment, and prompt identification of suspected cardiotoxicity are key in significantly reducing risk. This article provides a comprehensive review of the various types of treatment-related cardiotoxicity, offering guidance on identifying high-risk patients, recognizing early signs of cardiotoxicity, and outlining appropriate treatment approaches and follow-up care for such cases.

## 1. Introduction

In recent decades, there have been noteworthy advancements in chemotherapy, immunotherapy and targeted treatments, resulting in improved efficacy, tolerance and subsequently improved survival rates [1,2,3]. To maintain disease remission, it may be necessary to administer multiple cycles of treatment over an extended period, whilst additional lines of treatment are available, if required.

Despite these advancements, the potential for cardiotoxic effects persists. This issue becomes more significant in the presence of shared risk factors between oncologic and cardiovascular disease (CVD), such as smoking and a lack of exercise [3]. Patients with pre-existing CVD who undergo chemotherapy may be more susceptible to cardiotoxicity, consequently increasing morbidity and mortality risk within these populations [3]. It is important to note that despite revolutionizing modern oncology, even immunotherapy is not exempt from potential cardiac adverse events [4].

Cardiotoxic risk should be carefully evaluated in relation to the potential treatment benefits. However, by implementing early risk stratification, optimizing the management of pre-existing CVD, and promptly identifying cardiovascular complications, the risk can be minimized [5].

For risk stratification of cardiotoxicity from oncological treatment, the Heart Failure Association-International Cardio-Oncology Society (HFA-ICOS) risk assessment tool can be utilized, whilst overall CVD risk can be determined with scoring systems such as QRISK3, SCORE2/SCORE2-OP and ASCVD [5,6,7,8]. The HFA-ICOS scoring system takes into account factors such as prior cardiac history, previous cardiotoxic treatment, lifestyle, age, cardiac risk factors and cardiac biomarkers [5]. Patients are categorized accordingly into four groups: low, moderate, high, and very high risk, each with their specific surveillance and management guidelines.

In accordance with the European Society of Cardiology (ESC) cardio-oncology guidelines published in 2022, it is recommended to perform an electrocardiogram (ECG) for all patients scheduled to undergo cardiotoxic chemotherapy [9]. Additionally, transthoracic echocardiography (TTE) is routinely recommended for pre-treatment assessment of patients at a greater risk of cardiotoxicity. If any arrhythmias, pre-arrhythmic changes (such as QT prolongation), or pre-existing CVD are detected during the pre-treatment assessment, referral to a cardiology specialist is strongly advised (class Ia recommendation) as aggressive management of underlying disease is crucial for primary prevention.

This article provides a comprehensive review of the literature on various types of treatment-related cardiotoxicity, offering guidance on identifying high-risk patients, and recognizing early signs of cardiotoxicity, whilst outlining appropriate treatment approaches and emphasizing the importance of follow-up care.

## 2. Methods

Medline/PubMed and Google Scholar were searched from inception until July 2023 for publications in the English language reporting on cardiac toxicities in oncology. The search utilized (“Cardiotoxicity”[Mesh] AND “Anticancer Treatment”[Mesh]) in Medline, or keywords such as “cardiotoxicity”, “chemotherapy”, “immunotherapy”, “targeted therapy”, “heart failure” and “cardiovascular disease” in Google Scholar. We used the Cochrane Handbook for general steps for avoiding bias in screening studies for inclusion. Three—rather than two—reviewers worked independently to reduce the chance of error, to add more scrutiny and ensure proper conduct. The screening of articles was carried out manually by Y.S., A.B. and F.K., who assessed publication titles and abstracts for relevance. Among the articles retrieved, the reference lists of pertinent papers were also scrutinized to identify additional articles of interest for our review. Descriptive statistics were employed to summarize patient and therapeutic agent characteristics. IBM^©^ SPSS^©^ Statistics version 20 was utilized to compile and analyse the data, enabling us to provide an updated review of the literature on cardiac toxicities in oncology.

## 3. Common Cardiotoxicity Manifestations

### 3.1. Left Ventricular Dysfunction

Left ventricular ejection fraction (LVEF) decline and heart failure (HF) are common causes of premature interruption or discontinuation of sequential anthracycline chemotherapy [10]. Studies have reported that cardiac dysfunction can be exacerbated by the addition of trastuzumab, a human epidermal growth factor 2 (HER-2) monoclonal antibody, to existing anthracycline therapy [11,12]. Treatment-induced HF can be classified into two main categories: symptomatic and asymptomatic. Each category can be further classified based on the degree of symptom severity and LVEF. The LVEF values used for classification are as follows: LVEF ≤ 40% indicates HF with reduced ejection fraction (HFrEF), LVEF 41–49% indicates HF with mid-range ejection fraction (HFmrEF), and LVEF ≥ 50% indicates HF with preserved ejection fraction (HFpEF) [13].

Both the British Society of Echocardiography (BSE) and the British Society of Cardio-Oncology (BCOS) define cardiotoxicity as a decrease in LVEF by more than 10%, resulting in an LVEF value below 50% [14]. Additionally, the European Society of Medical Oncology (ESMO) interprets cardiotoxicity as a decline in LVEF exceeding 20% [15]. Moreover, probable subclinical cardiotoxicity can be characterized by an LVEF decline of more than 10%, resulting in a value above 50%, accompanied by a decrease in global longitudinal strain (GLS) exceeding 15% [16]. Possible subclinical cardiotoxicity, on the other hand, can be defined as an LVEF decline of less than 10%, leading to a value below 50%. Furthermore, an isolated reduction in GLS of more than 15% from baseline is also considered possible subclinical cardiotoxicity. In addition, brain natriuretic peptide (BNP) levels ≥ 35 pg/mL, NT-proBNP levels ≥ 125 pg/mL, or a significant increase from baseline can also serve as indicators of HF [17].

Anthracycline agents such as doxorubicin, have been widely used in chemotherapy regimens for almost five decades [18]. Doxorubicin inhibits the proliferation of cancer cells by interfering with the structure of their DNA, effectively impeding tumorigenesis and halting cancer cell division [19]. Anthracycline-induced cardiotoxicity is a well-known phenomenon that exhibits a dose-dependent relationship, culpable of irreversible HF with higher cumulative doses [20,21]. The estimated incidence rates are approximately 2% at a dose of 200 mg/m^2^, 5% at 400 mg/m^2^, 16% at 500 mg/m^2^, and 26% at 550 mg/m^2^ [22].

HER-2, encoded by proto-oncogene ErbB2 (chromosome 17q21-22), is a transmembrane protein with a molecular weight of 185 kDa [23]. Crucially, HER-2 regulates cell growth and epithelial cell survival, although overexpression and amplification of the HER-2 oncogene is typically observed in aggressive metastatic breast cancer, as well as other malignancies, such as ovarian, bladder, lung and head and neck [23]. Trastuzumab is a humanized monoclonal antibody that targets the HER-2 tyrosine kinase receptor [24]. LVEF is reduced by approximately 7%, but it can increase to 13% when trastuzumab is administered concurrently with paclitaxel, and up to 27% with concurrent anthracyclines [11]. Trastuzumab-related cardiac dysfunction is generally classified as “type II cardiotoxicity”, which is not dose-dependent and can be reversed upon discontinuation of the drug, often without significant ultrastructural changes [25]. Neuregulin (NRG) is an important stress-mediated paracrine growth factor that signals through the ErbB2 receptors to ensure cardioprotection [26]. However, trastuzumab promotes the inhibition of the NRG-1/ErbB2 signalling pathway within the heart, leading to apoptosis, oxidative stress, and T-tubule dilation, ultimately resulting in dilated cardiomyopathy [27].

Pertuzumab is another monoclonal antibody that inhibits the dimerization of HER2 receptors, which is an essential step required for cell growth and survival in several tumour types [28]. A pooled analysis of 569 patients treated with pertuzumab across different disease subsets revealed that 5.7% of patients experienced a decrease in LVEF, and 0.7% developed symptomatic congestive heart failure (CHF) [28]. In a phase II study evaluating the safety and efficacy of combined trastuzumab-pertuzumab treatment in 66 patients previously exposed to trastuzumab, asymptomatic LVEF reduction was observed in three patients, whilst no cases of CHF were reported [29].

Lapatinib is an oral small molecule that inhibits the tyrosine kinases of HER-2 and epidermal growth factor receptor type 1 (HER-1) [30]. Clinical studies did not report significant cardiotoxicity with the use of lapatinib (reference). A review of 44 clinical studies involving 3689 patients receiving lapatinib revealed a 0.2% rate of symptomatic CHF and a 1.4% rate of asymptomatic cardiac events [31]. Cardiac toxicity is typically asymptomatic and largely reversible, suggesting cellular dysfunction rather than myocyte damage [31].

Bcr-Abl inhibitors such as imatinib act by inactivating the Bcr-Abl fusion protein, which arises from a reciprocal chromosomal translocation between chromosome 9 and 22, known as t(9,22), resulting in the formation of the Philadelphia chromosome (Ph+) [32]. This fusion protein exhibits deregulated tyrosine kinase activity, leading to uncontrolled proliferation of myeloid precursors [32]. Imatinib was the first selective Bcr-Abl tyrosine kinase inhibitor (TKI) to be approved for chronic myelogenous leukaemia (CML) [33]. To overcome mutations and resistance, several second-generation TKIs have been developed and approved for clinical use, including nilotinib and dasatinib as first- or second-line treatments, and bosutinib as a second-line option [34]. Imatinib has been reported to induce cardiomyocyte death through the endoplasmic reticulum stress response, wherein the accumulation of misfolded proteins triggers apoptosis mediated by the c-Jun N-terminal kinase (JNK) pathway [35,36]. Studies monitoring imatinib cardiotoxicity suggest a very low incidence of HF, ranging between 0.2% and 1.7% [37]. However, the risk of HF has been reported to be higher in those with a history of heart disease, particularly CHF, hypertension, coronary artery disease, and cardiomyopathy [37]. In randomized controlled trials of patients with diagnosed chronic phase Ph+ CML, nilotinib has demonstrated better efficacy than imatinib, supporting nilotinib as a first line treatment option at either 300 mg or 400 mg twice daily [38,39].

Moreover, bortezomib is a proteasome inhibitor used in the treatment of multiple myeloma and mantle cell lymphoma [40]. By preventing targeted proteolysis, multiple signalling cascades are disrupted, ultimately causing apoptosis [40]. Acute development or exacerbation of CHF and new onset of decreased LVEF have been reported during bortezomib therapy, including in patients with no pre-existing risk factors [41].

Finally, bevacizumab acts by selectively binding circulating vascular endothelial growth factor (VEGF), inhibiting its binding to cell-surface receptors [42]. This inhibition restricts microvascular growth of tumour blood vessels, leading to a limitation in blood supply to tumour tissues [42]. These effects also reduce tissue interstitial pressure, increase vascular permeability, thus potentially enhancing the delivery of chemotherapeutic agents [42]. Although hypertension is a common side effect of bevacizumab, a reduction in LVEF has also been observed during long-term use [42].

### 3.2. QT Interval Prolongation

Several factors may contribute to QT interval prolongation. This includes commonly used medications in cancer patients such as chemotherapeutic agents (particularly TKI and arsenic trioxide therapies), antiemetics and antifungals [43]. Electrolyte abnormalities and myocardial ischemia are examples of other contributory factors that may prolong the QT interval [43,44]. It is important to note that QT prolongation can increase the risk of potentially life-threatening arrhythmias, such as torsades de pointes (TdP) [44].

Multitargeted TKIs have been associated with varying degrees of QT prolongation. Sunitinib, for example, has been shown to cause dose-dependent QT prolongation [45]. TdP, a polymorphic ventricular tachyarrhythmia, has been observed in less than 0.1% of patients receiving sunitinib [46]. Nilotinib is also known to prolong QTc interval, and there have been five reported cases of sudden cardiac death out of 867 patients treated in initial trials, leading to a warning on the United States Food and Drug Administration (US FDA) labelling [45,47]. The overall proportion of QTc prolongation of any grade with nilotinib was 2.7%, with a QTc interval greater than 500 ms observed in 0.3% of cases [48]. In the case of dasatinib, QT prolongation has been reported in <1% to 3% of patients, but the occurrence of a QTc interval greater than 500 ms was <1% [48].

Vandetanib is an orally administered selective inhibitor of VEGF receptor (VEGFR), epidermal growth factor receptor (EGFR), and RET tyrosine kinases [48]. A comprehensive meta-analysis of nine trials involving 2188 patients revealed that the overall incidence of all-grade and high-grade QTc interval prolongation with vandetanib at a dose of 300 mg/day was 16.4% (95% CI, 8.1–30.4%) and 3.7% (95% CI, 1.7–7.8%), respectively, among patients with non-thyroid cancer [48]. Among patients with thyroid cancer, the incidence was 18.0% (95% CI, 10.7–28.6%) for all-grade QTc interval prolongation and 12.0% (95% CI, 4.5–28.0%) for high-grade QTc interval prolongation [48].

Enzastaurin is a protein kinase C inhibitor that exerts its effects by suppressing the PI3K/Akt pathway, resulting in anti-angiogenic effects, inhibition of tumour growth, and induction of tumour cell death [49]. In a phase I trial involving 47 patients, three individuals experienced asymptomatic grade 3 QTc prolongation [46]. Additionally, in a combination phase I trial of enzastaurin with gemcitabine, one patient exhibited grade 2 QTc interval prolongation [50].

Vorinostat is a histone deacetylase inhibitor prescribed for the management of recurrent or persistent cases of cutaneous T-cell lymphoma [51]. A retrospective review of 116 patients participating in phase I and II clinical trials, who underwent baseline and follow-up ECGs, indicated that four patients experienced Grade 2 QTc interval prolongation, while one patient had Grade 3 prolongation. Notably, no cases of TdP were reported among patients treated with vorinostat [52].

Vemurafenib, a B-raf inhibitor, is prescribed for the treatment of patients with unresectable or metastatic melanoma carrying the V600E mutation of the B-raf protein [53]. A multicentre, open-label, single-arm study involving 132 patients with B-Raf V600E mutation-positive metastatic melanoma assessed the effects of twice-daily administration of vemurafenib at a dose of 960 mg on the QTc interval [54].

Pazopanib is an oral TKI that targets VEGFR-1, VEGFR-2, VEGFR-3, platelet-derived growth factor receptor (PDGFR), stem cell factor (SCF) and stem cell factor receptor (c-Kit) [55]. It is approved as a first-line treatment for advanced renal cell carcinoma [56]. Clinical studies investigating pazopanib have reported instances of QT interval prolongation and TdP arrhythmia, although this is considered very low risk (<1%) by a systematic review from Porta-Sanchez et al. [47].

### 3.3. Myocardial Ischemia

The fluoropyrimidines, 5-fluorouracil (5FU) and its oral prodrug capecitabine are frequently utilized in the treatment of gastrointestinal malignancies [57]. One well-documented complication associated with fluoropyrimidine therapy is coronary vasospasm, leading to myocardial ischemia (MI) and angina [57,58]. The reported incidence of cardiotoxicity with 5FU in the literature varies widely, ranging from 1% to 68% [58]. The toxicity appears to be influenced by both the dose and rate of administration, with higher doses (>800 mg/m^2^) and continuous infusion regimens being associated with elevated rates of toxicity [59].

The primary cardiotoxicity associated with taxanes is bradycardia, although ischemia has also been reported [60]. The incidence of cardiotoxicity with paclitaxel is reported to range between 0.5% and 5%, whilst with docetaxel, it is approximately 1.7% [46]. The exact mechanism underlying this cardiotoxicity is not well-defined [46].

A pooled analysis of 1745 patients enrolled in five randomized controlled trials involving colorectal, non-small cell lung cancer and metastatic breast cancer reported an incidence of angina and MI of 1.5% in the bevacizumab group [61]. In another meta-analysis investigating a total of 4617 patients from seven randomized controlled trials, the summary incidence of ischemic heart disease in patients receiving bevacizumab was 1.0% (95% CI, 0.6–1.4%) [62]. The analysis also revealed that patients treated with bevacizumab had a significantly increased risk of ischemic heart disease with a relative risk (RR) of 2.49 (95% CI, 1.37–4.52) compared to the control arm [62].

Sorafenib is a multi-targeted inhibitor that acts on tyrosine kinase receptors, including c-Kit, Flt-3, PDGFR-b, VEGFR-2 and VEGFR-3, as well as serine/threonine kinases B-Raf and Raf-1 [63]. It is approved for the treatment of advanced renal cell carcinoma, refractory differentiated thyroid carcinoma, and hepatocellular carcinoma [64]. According to an independent review from the FDA, there is a higher incidence of cardiac ischemia observed in patients treated with sorafenib compared to the placebo group (2.9% vs. 0.4%) [65]. For renal cell carcinoma specifically, MI or ischemia were more prevalent than in the control group (3% vs. <1%) [65].

### 3.4. Hypertension

Systemic anti-cancer treatment has been identified as a potential cause of secondary hypertension, and the use of bevacizumab, as mentioned previously, is often associated with an increased incidence or worsening of existing arterial hypertension [42]. The leading hypothesis for the mechanism of bevacizumab-induced hypertension is the inhibition of VEGF-mediated vasodilation, resulting in increased vascular tone [66]. Other proposed mechanisms include a reduction in capillary density, leading to elevated systemic vascular resistance and pressure in larger vessels, as well as alterations in renal function due to the role of VEGF in maintaining normal glomerular structure and filtration in kidney endothelial cells and podocytes [66]. In a comprehensive meta-analysis comprising 12,656 patients with various types of tumours across 20 studies, the incidence of all-grade hypertension in patients receiving bevacizumab was found to be 23.6%, with 7.9% classified as high-grade (grade 3 or 4) [67]. Notably, patients treated with bevacizumab were at a significantly increased risk of developing high-grade hypertension, with a RR of 5.28 compared to controls [67].

Multitargeted TKIs such as sunitinib, sorafenib, and pazopanib exert their anti-angiogenic effects by inhibiting the catalytic binding site on VEGFR2, and as a result, they are also known to induce hypertension through similar mechanisms [68]. In a comprehensive systematic review and meta-analysis encompassing 4999 patients with renal cell carcinoma and other malignancies from 13 clinical trials, the incidence of all-grade and high-grade hypertension among patients receiving sunitinib was found to be 21.6% and 6.8%, respectively [69]. Additionally, sunitinib treatment was significantly associated with a higher risk of high-grade hypertension and renal dysfunction compared to the control arm [69]. Similar findings were observed for sorafenib in another extensive systematic review and meta-analysis that included 4599 patients with renal cell carcinoma or other solid tumours [70]. Among these patients, the overall incidence of all-grade and high-grade hypertension was 23.4% (95% CI 16.0–32.9%) and 5.7% (2.5–12.6%), respectively [70]. Sorafenib treatment was significantly associated with an increased risk of all-grade hypertension in cancer patients, with a RR of 6.11 compared to controls [70]. Finally, in a small study of 35 patients, pazopanib-induced hypertension was observed in 57% of the participants [71].

### 3.5. Thromboembolic Disease

In comparison to chemotherapy alone, the combination of bevacizumab and chemotherapy has been found to carry an increased risk of arterial thromboembolism (ATE), while the risk of venous thromboembolism (VTE) remains unaffected [72]. It is believed that bevacizumab may increase the risk of arterial thrombotic events by interfering with the regeneration process of endothelial cells following incidental trauma during anti-VEGF treatment [73]. The incidence of ATE was 5.5 events per 100 person-years among those receiving combination therapy, compared to 3.1 events per 100 person-years in the chemotherapy alone group [72]. A retrospective pooled analysis of five randomized trials further confirmed the association between bevacizumab use and arterial thromboembolic events, with a higher incidence of 3.8% compared to 1.7% in patients receiving chemotherapy alone [72]. Age ≥65 and a prior history of ATE were identified as risk factors associated with the occurrence of ATE in patients treated with bevacizumab [74].

Furthermore, several VEGFR-TKIs have been found to be associated with an increased risk of thromboembolic disease [75]. A comprehensive meta-analysis, including 24,855 patients from 48 studies, revealed an overall incidence of 3.6% for all-grade VTE and 1.6% for high-grade VTE in association with VEGFR-TKIs [73]. However, the use of VEGFR-TKIs did not significantly increase the risk of developing VTE [73]. Regarding arterial thromboembolic events, the overall incidence of all-grade ATE was 2.7%, and high-grade ATE was 0.6% among patients receiving VEGFR-TKIs [73]. The use of VEGFR-TKIs was found to significantly increase the risk of developing all-grade arterial thromboembolic events, with a tendency to also increase the risk of high-grade ATE [73]. Therefore, patients with cancer who receive VEGFR-TKIs are at a higher risk of developing ATE [73]. Moreover, a systematic review and meta-analysis involving 10,255 patients from January 1966 to July 2009 reported the incidence of ATE associated with the use of sunitinib and sorafenib to be 1.4%, with the RR for ATE compared to control patients calculated as 3.03 [76].

Thalidomide and lenalidomide, immunomodulatory drugs that exhibit anti-angiogenic properties, have also demonstrated a high risk of VTE, particularly when combined with high-dose dexamethasone [77]. They are also associated with an increased risk of arterial events, leading to a concern of an elevated risk of MI and stroke [78].

### 3.6. Valvular Heart Disease

While chemotherapeutic agents do not directly impact cardiac valves, valvular heart disease (VHD) can occur in cancer patients due to various factors [79]. These include pre-existing valve lesions, radiotherapy (RT), infective endocarditis, and VHD secondary to left ventricular dysfunction (LVD) [79,80]. Among these, approximately 10% of patients will frequently experience RT-induced VHD, which is characterized by fibrosis and calcification, primarily affecting the aortic root, aortic valve cusps, mitral valve annulus, and the base and mid portions of the mitral valve leaflets [79]. Patients with Hodgkin lymphoma are particularly at risk, as higher radiation doses (>30 Gy) to the heart valves can significantly increase the likelihood of clinically significant VHD [81]. This represents a common cardiovascular complication following treatment.

### 3.7. Pericardial Disease

RT-induced pericarditis can manifest early, but studies have shown that the peak incidence occurs between 5 and 9 years post-RT in patients with breast cancer, with the risk and severity of pericarditis influenced by the radiation dose [82,83]. In a review, more than 50% of patients who received doses exceeding 30 Gy for various diseases, such as Hodgkin and non-Hodgkin lymphoma developed pericarditis [84]. In addition, studies have specifically investigated the correlation between the extent of RT to the heart and the development of RT-induced pericarditis, concluding that a larger proportion of the heart exceeding 30%, receiving a minimum dose of 50 Gy, increases the likelihood of pericarditis [85]. It is important to note that pericardial effusion can occur as a complication of pericarditis, but it can also be secondary to pericardial metastasis, which, although rare, could be the initial indication of an underlying malignancy [86].

Acutely following the administration of anthracyclines, myopericarditis can occur [21,87]. Furthermore, high-dose cyclophosphamides have been associated with acute cardiotoxicity, presenting as haemorrhagic myopericarditis [88,89]. In the context of treating acute promyelocytic leukaemia, the use of all-trans retinoic acid (ATRA) has also been reported to induce acute myopericarditis [90].

### 3.8. Cardiac Device Malfunction

A comprehensive retrospective study by Zagzoog et al. analysed data from 811 patients who underwent RT, and among them, device malfunctions were observed in 32 patients (4%) [91]. A notable association was found between higher beam energy (≥10 MV) and device malfunction (*p* < 0.0001) [91]. However, there was no significant difference in the dose of the RT between the group experiencing malfunctions and the group without [91].

## 4. Immunotherapy and Cardiotoxicity

Immune checkpoint inhibitors (ICIs) such as ipilimumab (anti-cytotoxic T-lymphocyte–associated antigen 4 (anti-CTLA-4)) and nivolumab/pembrolizumab (anti-programmed death-1 (anti-PD-1)) have demonstrated significant tumour responses in various cancer types, including melanoma, non-small cell lung cancer, renal cell cancer, and Hodgkin’s lymphoma [92,93]. While cardiovascular immune-related adverse events (irAEs) are rare, they can be life-threatening [93]. A meta-analysis of 26 studies involving 4622 ICI-treated cancer patients reported myocarditis in 0.5%, HF in 0.3%, and atrial fibrillation in 4.6% of patients [93]. Pericardial effusion, cardiomyopathy, MI, and cardiac arrest were observed in 0.5%, 0.3%, 0.4% and 0.4% of patients, respectively [93]. It is believed that T-lymphocyte-mediated inflammation can injure the myocardium, affecting cardiac remodelling and therefore function [94]. Combination immunotherapy has been identified as a risk factor of cardiovascular irAEs [93]. Data from the Bristol-Myers Squibb safety database demonstrated an estimated myocarditis rate of 0.27% in patients receiving combination immunotherapy of ipilimumab plus nivolumab compared to 0.06% in those receiving nivolumab monotherapy [95].

## 5. Management

### 5.1. QT Prolongation

QTc prolongation is associated with a higher risk of mortality in cancer patients, and therefore, it is crucial to promptly recognize and manage it effectively [43]. To achieve this, it is recommended to obtain an ECG prior to treatment, enabling periodic assessments of the QTc interval [47]. According to the ESC guidelines, a follow-up ECG should be performed two weeks after starting or increasing the dose of treatment, followed by monthly ECGs for the first three months [9]. However, patients undergoing arsenic trioxide treatment require weekly monitoring of their QT interval, as studies have shown that up to 40% of individuals on arsenic trioxide will experience a QTc interval exceeding 500 ms [9,47].

The primary emphasis of treatment should be on evaluating and rectifying electrolyte imbalances, as chemotherapy-related adverse effects include diarrhoea and vomiting [96,97]. This is crucial in preventing severe complications, such as TdP. Moreover, it is essential to carefully review certain medications that may prolong the QTc interval, such as macrolides, selective serotonin reuptake inhibitors and some anti-emetics [98]. A QTc interval exceeding 500 ms or a QTc interval increase of more than 60 ms from baseline is of particular concern, and according to the US FDA and Drug Administration and the European Medicines Agency, it is recommended to temporarily discontinue anti-cancer therapies [99]. Additionally, the occurrence of symptoms such as syncope alongside a prolonged QTc interval should be taken seriously, necessitating urgent medical evaluation and temporary suspension of oncology treatment [99]. Once the QTc interval returns to baseline, treatment may be resumed, but at a lower dosage and with closer monitoring due to increased susceptibility to QTc prolongation [99]. In the event that TdP occurs, intravenous magnesium sulfate remains the primary treatment approach [99]. The advanced life support algorithm should be followed, and if cardiac arrest occurs, non-synchronized defibrillation should be performed [99]. Moreover, in cases of decreased cardiac output, if the rhythm does not respond to magnesium therapy, overdrive pacing or isoprenaline can be considered to achieve a heart rate above 90 beats per minute [99].

### 5.2. Myocardial Ischemia

Before initiating oncological therapies, it is crucial to determine whether there is pre-existing coronary artery disease. Specifically, the use of fluoropyrimidines, cisplatin, VEGF inhibitors, and RT increase the risk of myocardial ischemia and infarction [99,100]. If ischemia is determined to be a result of the oncological treatment, the medication responsible should be discontinued, and an alternative class of antineoplastic drugs should be considered [100]. The initial diagnostic evaluation follows the same approach as for non-cancer patients [99,100,101]. Therefore, TTEs continue to play an important role, followed by assessment for ischemia through either anatomical or functional testing [99].

The management of these patients necessitates a collaborative approach involving a multidisciplinary team (MDT) consisting of oncologists and cardiologists. This approach involves assessing the long-term cancer prognosis, bleeding risk, planned future anti-cancer treatment, and risk of thrombotic events [99,101]. The first-line medical treatment for angina focuses on antianginal therapies, which offer both symptomatic relief and prognostic benefits [99]. Considering the vasospastic mechanisms of angina associated with oncological therapies, both oral nitrates and non-dihydropyridine calcium channel blockers play a significant role [99,101]. The use of antiplatelets or anticoagulation in this specific patient population poses a clinical dilemma, as oncological therapies may result in bone marrow suppression and impaired liver function, leading to thrombocytopenia [101,102]. Consequently, thrombocytopenia significantly increases the risk of bleeding, with the severity of thrombocytopenia correlating with the bleeding risk [101,102,103]. Notably, the risk of spontaneous bleeding rises exponentially when the platelet count falls below 10 × 10^9^/L [103]. However, small retrospective observational studies on cancer patients with thrombocytopenia who develop acute coronary syndromes suggest that aspirin dramatically improves survival without increasing the risk of major bleeding [104,105,106]. Therefore, assessing platelet function rather than platelet count becomes essential in tailoring antiplatelet therapies appropriately for each individual patient. Based on the available data, the Society for Cardiac Angiography and Interventions (SCAI) recommends initiating aspirin once the platelet count exceeds 10,000/mL and adding clopidogrel when the platelet count is 30,000–50,000/mL [107].

Thrombocytopenia may pose challenges in performing coronary angiography and subsequent percutaneous coronary intervention (PCI) [108]. A study by Darcy et al. revealed that among 1000 patients undergoing femoral angiography, those with platelet counts below 100 × 10^9^/L were significantly more likely to develop hematomas [109]. Therefore, in such patients, radial access should be preferred over femoral access due to its superior safety profile [110]. Additionally, data from multi-centre registries has shown that, in cancer patients with ischemic heart disease, PCI reduces all-cause mortality compared to those who do not undergo PCI [111]. However, the available data does not provide a breakdown of the number of patients with chronic coronary syndrome or acute coronary syndrome, nor does it provide details regarding optimal medical therapy [111].

### 5.3. Hypertension

Similar to the general population, hypertension in patients with malignancy is defined as a blood pressure reading exceeding 140/90 mmHg [112]. However, according to the guidelines of the American College of Cardiology (ACC) and the American Heart Association (AHA), a lower blood pressure threshold of 130/80 mmHg should be used [113]. It is crucial to assess other pre-existing cardiovascular risk factors and proteinuria to determine the clinical risk associated with hypertension [114]. Consensus documents state that unless there is a hypertensive emergency or end-organ damage due to hypertension, oncological therapies should be continued [115]. The main treatment approach includes the use of angiotensin-converting enzyme (ACE) inhibitors, angiotensin receptor blockers (ARBs), and dihydropyridine calcium channel blockers [115]. However, the combination of non-dihydropyridine calcium channel blockers like verapamil and diltiazem with VEGF inhibitors should be avoided due to their interaction with the cytochrome P450 3A4 enzyme, which can lead to toxic levels of VEGF inhibitors [115]. Specifically, beta-blockers should be considered in cancer patients at an increased risk of developing LVD, such as those receiving anthracycline therapies [116]. Diuretics should be used cautiously to avoid dehydration and electrolyte imbalances, which may be exacerbated by ongoing cancer treatment [117,118]. However, it is important to acknowledge the limited evidence available regarding the effects of specific antihypertensive medications and their outcomes in reducing blood pressure in cancer patients, as most of the data is derived from retrospective observational cohort studies.

### 5.4. Heart Failure

Identifying patients who are at the highest risk of developing HF is crucial, as it is widely acknowledged as one of the most prevalent forms of cardiotoxicity [119]. This involves conducting a thorough evaluation of cardiovascular risk factors and recognizing the chemotherapeutic agents commonly associated with that risk.

Current evidence suggests that baseline cardiac biomarkers and TTE have a role in identifying those who are at risk of experiencing major adverse cardiovascular events (MACE) [120]. While the use of troponin in this patient population is not well-established, a prospective study of 703 patients undergoing high-dose anthracycline chemotherapy, found that consistently elevated troponin levels before and after chemotherapy were significantly associated with the development of severe left ventricular systolic dysfunction (LVSD) and MACE three years post-treatment [121]. It is noteworthy that there were five deaths in the troponin-positive group compared to none in the troponin-negative group [121]. Similar adverse findings have been observed in breast cancer patients receiving trastuzumab, with elevated baseline troponin levels predicting a 2.4 to 4.5-fold increased likelihood of developing LVSD (*p* < 0.001) [122]. Additionally, meta-analyses have shown a negative predictive value of 93% for troponin in detecting cardiotoxicity, indicating its value as a screening tool to identify those at risk [123].

The role of natriuretic peptides in predicting the onset of cardiotoxicity remains unclear. Limited studies and meta-analyses suggest no correlation between higher natriuretic peptide levels and chemotherapy-induced LV dysfunction [124]. It is important to note that due to the observational nature and small size of the studies investigating cardiac biomarkers, the ESC guidelines provide different recommendations regarding their utility based on the type of oncology treatment [9]. For anthracycline chemotherapy-induced cardiotoxicity, troponin level monitoring is a Class I recommendation for high-risk patients before each cycle and at 3 and 12 months post-chemotherapy, and a Class IIa recommendation for moderate-risk patients. For HER-2 related cardiotoxicity, troponin monitoring is a Class IIa recommendation for high-risk patients and a Class IIb recommendation for low- to moderate-risk patients.

TTEs play an important role in detecting LVSD, and it is recommended to perform a baseline TTE in those scheduled to receive anthracycline or HER-2 therapy [14]. If the initial TTE reveals a reduced LVEF of <50%, cardioprotective therapies should be initiated, and referral to specialized cardio-oncology services should be considered for alternative non-cardiotoxic cancer treatments [15]. The PRADA (Prevention of Cardiac Dysfunction during Adjuvant Breast Cancer Therapy) trial provides contemporary evidence supporting the prophylactic use of candesartan to prevent a decline in LVEF in patients treated with high-dose anthracyclines with or without trastuzumab and RT [125]. Moreover, data from a single-centre randomized controlled trial in Spain involving 90 patients suggested that a combination of enalapril and carvedilol significantly prevents LVSD and death in patients with haematological malignancies undergoing high-dose chemotherapy [126]. In HER-2 positive breast cancer patients receiving trastuzumab, both lisinopril and carvedilol reduced the incidence of cardiotoxicity by 47% and 51%, respectively, compared to placebo after 2 years of follow-up, in addition to concomitant anthracycline therapy [127]. Mineralocorticoid receptor antagonists (MRAs), such as spironolactone, have also shown significant benefits in preventing LVEF reduction in breast cancer patients treated with anthracyclines compared to placebo [128]. These findings are supported by meta-analyses that demonstrate a favourable effect on LVEF with the use of ACE inhibitors, ARBs, beta-blockers, and MRAs as primary prevention in cancer patients undergoing anthracycline or HER-2 therapies [129,130,131]. However, there are no significant differences observed in clinical outcomes such as HF symptoms or mortality [129,130,131]. Therefore, contemporary guidelines provide a Class IIa recommendation for the use of ACE inhibitors or ARBs and beta-blockers as primary prevention therapy in high-risk cancer patients receiving oncological therapies that may increase the risk of HF [9].

Additionally, dexrazoxane, an iron chelator, may also be considered for cardioprotection to effectively reduce toxicity caused by free radicals from high-dose anthracycline therapy [9,132]. The current 2022 ESC guidelines recommend the use of dexrazoxane as a Class IIa recommendation for high-risk patients scheduled to undergo anthracycline chemotherapy [9]. Table 1 depicts cardiovascular risk factors and the relevant chemotherapeutic agents commonly associated with this significant issue, whereas Table 2 provides recent recommendations for surveillance of heart complications for patients on anthracycline and anti-HER2 treatments.

## 6. Prevention

### 6.1. Molecular and Cellular Biomarkers

In addition to the established cardiac biomarkers, troponin and natriuretic peptides, emerging biomarkers including myeloperoxidase (MPO), microRNA, Galectin-3, and growth differentiation factor-15 (GDF-15) show promise in detecting subclinical cardiotoxicity [135,136]. Specifically, MPO has been shown to be predictive of cardiotoxicity in breast cancer patients receiving doxorubicin and trastuzumab [137]. Patients who experienced an increase in MPO levels from baseline to 3 months had a 34% higher risk of developing LVD [137]. Furthermore, a meta-analysis involving 1979 patients, predominantly with breast cancer (99%) receiving anthracycline treatment, demonstrated that elevated MPO levels after treatment were associated with an increased risk of cardiotoxicity [138]. Interestingly, the study concluded that elevated MPO levels were a more effective indicator of treatment response than troponin or natriuretic peptides. Additionally, both GDF-15 and Galectin-3 have been associated with the development of early anthracycline-induced cardiotoxicity [139]. Specifically, a doubling of both GDF-15 and Galectin-3 levels 3 months after completing anthracycline therapy was linked to a 300% and 400% increased risk of cardiotoxicity, respectively [139].

MicroRNAs have emerged as potential markers of cardiac damage due to their role in regulating fundamental cardiac processes [140,141]. Elevated levels of microRNAs have been associated with cardiac pathologies such as HF, MI, atherosclerosis and hypertension. Cardiomyocyte-specific microRNAs, including MiR-29b and MiR-499, have shown increased expression within 6 to 24 h after anthracycline therapy, with the magnitude of elevation correlating with traditional markers of myocardial injury [142]. A systematic review conducted by Brown et al. identified 11 useful microRNAs (miR-1, 17, 19a, 29a, 34a, 122, 130a, 199a, 378a, 423 and 499) for identifying cardiac damage in breast cancer patients undergoing chemotherapy [143]. However, the review highlighted the limitations of microRNA studies, such as small sample sizes and significant variation in endpoints and definitions of cardiotoxicity. Therefore, further longitudinal studies are needed to validate the utility of these biomarkers in predicting subclinical cardiotoxicity more comprehensively.

**Figure 1:**
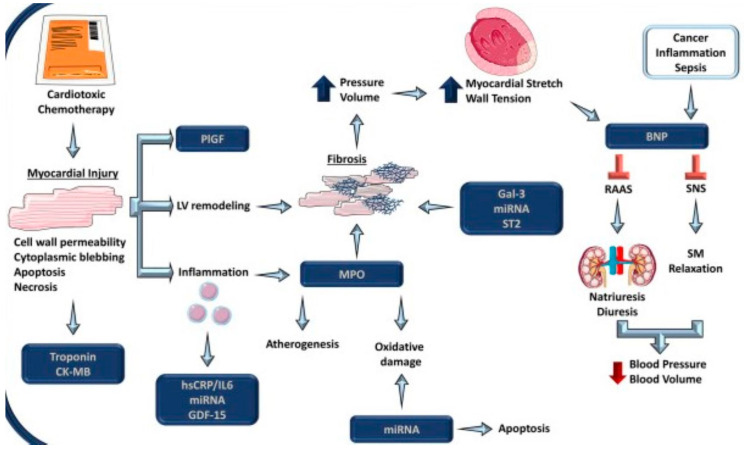
Various biomarkers play a crucial role in assessing cardiac damage caused by cancer therapy. These biomarkers reflect different aspects of myocardial health, including myocardial injury (troponin, CK-MB, GDF-15), stretch and stress (BNP), inflammation (hsCRP, IL-6, GDF-15), oxidative stress (MPO, miRNA), fibrosis (MPO, gal-3, miRNA, ST2) and angiogenesis (PlGF). Among these, troponin and BNP are the most frequently utilized in cardiac assessments. Troponin indicates myocardial damage, whereas BNP indicates stress that leads to neurohormonal changes, ultimately inhibiting the renin-angiotensin system (RAAS) and sympathetic nervous system (SNS). Created with BioRender.com, accessed on 12 October 2023.

Figure 1 illustrates the pathophysiology of biomarkers and their corresponding mechanisms in the context of general cardiovascular disease.

### 6.2. Molecular Cardiac Imaging

Molecular nuclear imaging holds great promise in the prediction and early detection of cardiotoxicity, aiming to prevent its devastating consequences [144]. Specifically, anthracycline-induced cardiotoxicity is associated with increased levels of free radicals, and a unique positron emission tomography (PET) tracer, known as [18F]-6-(4-((1-(2-fluoroethyl)-1H-1,2,3-triazol-4-yl)methoxy)phenyl)-5-methyl-5,6 dihydrophenanthridine-3, 8-diamine ([18F]·DHMT), is able to detect them [144]. In an animal model study comparing rats with doxorubicin-induced cardiotoxicity to controls, elevated levels of 18F-DHMT were observed two weeks prior to the onset of LVD, suggesting its potential in predicting cardiotoxicity [145]. Furthermore, free radicals may trigger pathological cardiac remodelling by activating matrix metalloproteases (MMPs), which can be detected using a single-photon emission computed tomography (SPECT) radiotracer, 99mTc-RP805 [146,147]. Animal studies have shown a correlation between elevated MMP levels and 99mTc-RP805, which is associated with a significant reduction in LV function [148]. Another PET tracer, 68Ga-Galmydar, is valuable in detecting the detrimental effects of free radicals by identifying mitochondrial dysfunction caused by them [149]. In one study, rodents injected with doxorubicin exhibited a two-fold decrease in 68Ga-Galmydar uptake on PET-CT scans 5 days after treatment, compared to control subjects [149]. This reduction was associated with cellular apoptosis and subsequent doxorubicin-induced cardiotoxicity [149]. In human studies, increased myocardial uptake of 111In-antimyosin and decreased uptake of 123I-metaiodobenzylguanidine (MIBG) as anthracycline doses escalate have been associated with the onset of cardiotoxicity [150].

PET radiotracers that target inflammatory cells such as CD4+, CD8+, and fibroblast activating protein (FAP), may play a crucial role in the early detection of subclinical myocarditis [144]. Specifically, ^68^Ga-FAPI uptake, a marker for FAP, has shown significant elevation in patients with ICI-related myocarditis compared to non-myocarditic patients [151]. Promising markers such as ^89^Zr-DFO-CD4 and ^89^Zr-DFO-CD8a, which target CD4+ and CD8+ T cells, respectively, have the potential to identify very early histological myocarditis before the onset of clinical symptoms [152].

In addition to nuclear imaging, cardiac magnetic resonance imaging (MRI) techniques using hyperpolarized carbon-13 can be employed to assess the status of oxidative phosphorylation, which is impaired in anthracycline-induced cardiotoxicity [144]. Animal studies have demonstrated the ability of hyperpolarized cardiovascular magnetic resonance to detect mitochondrial dysfunction and identify anthracycline-induced cardiotoxicity prior to the development of clinical symptoms [153]. While further large-scale studies are needed for both PET nuclear imaging and cardiac MRI, these findings underscore the importance of utilizing a multimodal approach to detect early cardiotoxicity before functional deterioration occurs.

### 6.3. Identification of High-Risk Populations

**Figure 2:**
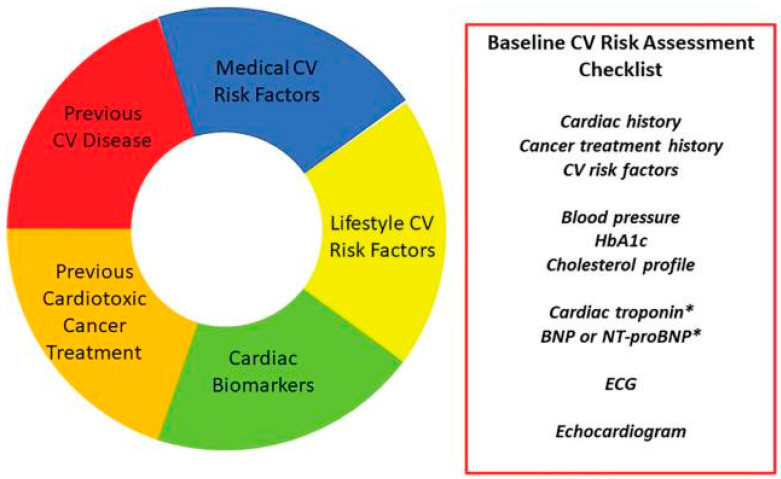
Various risk factors contribute to the initial cardiovascular (CV) risk in a cancer patient. A checklist encompassing clinical history and essential baseline investigations is necessary before initiating such therapy. * It is recommended to measure cardiac biomarkers like troponin and natriuretic peptides, if accessible [5]. Created with BioRender.com, accessed on 12 October 2023. Key abbreviations: BNP, brain natriuretic peptide; ECG, electrocardiogram; HbA1c, glycated haemoglobin; NT-proBNP, N-terminal pro-brain natriuretic peptide.

When considering potential cardiotoxic oncological therapies and the need for meticulous cardio-oncology surveillance, it is crucial to conduct a baseline cardiovascular risk assessment for all patients. To facilitate this assessment, HFA-ICOS has developed a risk stratification tool, which encompasses various categories of risk assessment, including pre-existing CVD, elevated cardiac biomarkers, comorbidities, demographic variables associated with cardiovascular risk, previous exposure to cardiotoxic cancer therapies, and lifestyle-related cardiovascular risk factors [5]. This tool and its components are illustrated in Figure 2. Each factor is assigned a specific level of risk based on the corresponding oncological therapy. By completing the risk assessment proforma, patients can be classified into one of four risk categories: low-, medium-, high- or very high-risk. These categories correspond to future cardiotoxicity risks of 2%, 2–9%, 10–19% and >20%, respectively [5]. Furthermore, specific recommendations are provided for each risk class.

1. Patients classified as low-risk should proceed with their oncological treatment and adhere to the cardiovascular surveillance protocols outlined in local or national guidelines [5].
2. Patients categorized as medium-risk should receive enhanced monitoring of their cardiovascular risk, with careful consideration given to the possibility of referral to a specialized cardio-oncology centre [5].
3. Patients identified as high- or very high-risk should be referred for a comprehensive cardio-oncological assessment. This assessment will help determine the suitability of alternative cancer therapies and allow for a personalized approach to their ongoing cardiovascular follow-up, taking into account their specific needs and circumstances [5].

It is crucial to prioritize timely completion of the baseline cardiovascular risk assessment to avoid any unnecessary delays in critical cancer therapies. In emergency situations, it is advisable to initiate oncological treatments promptly, deferring the baseline cardiovascular risk assessment until the patient’s stability is ensured. However, it is important to note that potentially curative cancer treatments should only be withheld in patients at high or very high risk of developing cardiotoxicity after thorough MDT discussions involving oncologists, haematologists, and cardiologists. Furthermore, ongoing validation of the HFA-ICOS risk stratification proforma is necessary to evaluate its effectiveness in reducing adverse cardiovascular and cancer outcomes. This validation process will help ensure its reliability and utility in clinical practice.

## 7. Conclusions and Future Direction

With the continuous expansion of oncological therapies, the inevitable cardiotoxic effects of these medications must be acknowledged and identified. Therefore, ongoing validation of the HFA-ICOS risk stratification tool for baseline risk assessment is essential, along with extrapolating outcome data to evaluate its effectiveness in reducing cardiotoxicity. Furthermore, it is imperative to conduct further longitudinal and prospective research to determine the most optimal and effective prophylactic therapies for preventing myocardial and endothelial damage caused by cardiotoxic therapies. Additionally, there is a notable gap in the literature regarding follow-up care for cancer survivors who have completed their oncological regimen. Therefore, both imaging and biomarker parameters should be incorporated in the evaluation of this specific patient population to better understand the long-term effects of these medications on their cardiovascular health.

Promoting education and raising awareness among medical practitioners about the potential cardiotoxic effects of cancer therapies is a crucial aspect in mitigating adverse outcomes. It enables early identification of high-risk patients and facilitates prompt involvement of specialists, ensuring that patients can undergo their cancer treatment with appropriate cardiovascular care. Cardio-oncology represents a dynamic and rapidly advancing field within cardiology. However, the collaborative efforts of an MDT consisting of oncologists, haematologists, and cardiologists are pivotal. Together, they thoroughly assess each case, allowing for a personalized approach to cardioprotective therapies, surveillance, and consideration of suitable non-cardiotoxic alternatives. By adopting this approach, superior outcomes can be achieved, optimizing both cancer treatment and cardiovascular health.

## Figures and Tables

**Table 1:** Patient- and therapy-associated risk factors of anthracycline cardiotoxicity.

Patient-Associated Risk Factors	Therapy-Associated Risk Factors
Female gender [21]	Higher individual anthracycline doses and a cumulative dose of anthracycline > 350 mg/m^2^ [21]
Age > 65 years [133]	Previous cardiotoxicity secondary to anthracyclines or trastuzumab [21,133]
History of hypertension, smoking, obesity, hyperlipidemia, diabetes mellitus [133,134]	Prior mediastinal radiation therapy [21]
History of cardiac disease [21,133]	Concomitant treatment with cyclophosphamide, trastuzumab or paclitaxel [21]

**Table 2:** ESC 2022 guidelines for surveillance of heart complications following anthracycline/anti-HER2 treatment [9].

Therapy	Recommendations	
Anthracycline	A baseline ECG and TTE are recommended for all patients prior to starting treatment (class I). TTE is recommended within 12 months after completing treatment for all patients (class I). Baseline cardiac biomarkers (cTn and NP) are recommended for high-/very high-risk patients at baseline (class I) and can also be considered prior to treatment for low-/moderate-risk patients (class IIa).	
	Low/Moderate Risk: An additional TTE should be considered after reaching a cumulative dose of >250 mg/m^2^ of doxorubicin or equivalent in low-risk (class IIb) and moderate-risk (class IIa) patients. cTn and NP monitoring every two cycles and within 3 months of therapy completion for patients receiving a cumulative dose of >250 mg/m^2^ of doxorubicin or equivalent (class IIa).	High/Very High Risk: TTE should be performed every two cycles during treatment and within 3 months after completing treatment (class I). cTn and NP monitoring before every cycle and 3 and 12 months after treatment completion (class I).
Anti-HER2	A baseline ECG and TTE is recommended for all patients before initiating treatment, followed by TTEs every 3 months during treatment and at 12 months after completing treatment (class I).	
	Low/Moderate Risk: In low-risk, asymptomatic patients with normal assessment after 3 months, consider reducing TTEs to every 4 months (class IIb). Consider baseline cTn and NP monitoring at baseline, every 3 months and 12 months after therapy in low-/moderate-risk patients (class IIb).	High/Very High Risk: Baseline cTn and NP are recommended for high-/very high-risk patients prior to treatment (class I) and consider monitoring every 2–3 cycles during therapy and 3 and 12 months following treatment (class IIa).

Abbreviations: cTn, cardiac troponin; NP, natriuretic peptide; ECG, electrocardiography; TTE, transthoracic echocardiography; anti-HER2, anti-human epidermal growth factor receptor 2.

## Data Availability

No new data were created or analysed in this study. Data sharing is not applicable to this article.
